# Carbonation Treatments for Durable Low-Carbon Recycled Aggregate Concrete

**DOI:** 10.3390/ma18174168

**Published:** 2025-09-05

**Authors:** Ruth Saavedra, Miren Etxeberria

**Affiliations:** Department of Civil and Environmental Engineering, Campus Nord, Universitat Politècnica de Catalunya·Barcelona TECH, 08034 Barcelona, Spain; ruth.saavedra@upc.edu

**Keywords:** CO_2_ curing process, carbonated recycled aggregates concrete, low-carbon cement, compressive strength, durability

## Abstract

The use of supplementary cementitious materials and the CO_2_ uptake capacity of cementitious materials—including recycled concrete aggregates—not only promotes the circular economy but may also present an opportunity to increase their ecoefficiency, thus improving the shrinkage and durability properties of concretes. This study analyses the impact of carbonated recycled aggregates and CO_2_ curing on improving the properties of commercial structural self-compacting concrete. Recycled aggregate concretes (RACs) were produced using 50% and 60% coarse recycled concrete aggregate (RCA), in carbonated and uncarbonated forms, and two types of cement—ordinary Portland cement (CEM I) and CEM II/B-M Portland composite cement containing 24% less clinker than CEM I—all with similar compressive strengths. After evaluating the CO_2_ curing process, the physical, mechanical, shrinkage, and durability properties (including suction and carbonation resistance) of the concretes were assessed. The properties of the RACs were compared with those achieved by conventional concrete, to generate insights for developing a highly sustainable concrete manufacturing process. Taking all the assessed properties into account, the CO_2_ curing process improved concrete’s properties. In addition, RAC-C50-I concrete (using CEM I with carbonated RCA) and RAC50-II (using CEM IIB and uncarbonated RCA) exhibited the greatest durability, resulting in reductions in sorptivity values of 40% and 45%, and decreases in the carbonation coefficient of 16% and 21%, respectively, compared to concrete without CO_2_ curing.

## 1. Introduction

The activities involved in producing cementitious materials contribute approximately 7–8% of global CO_2_ emissions [1,2]. However, only 8% of the total CO_2_ emissions from cementitious materials is attributed to the extraction and utilisation of aggregates. Thus, the incorporation of recycled aggregates promotes the circular economy and facilitates the production of highly sustainable construction materials by reducing natural resource consumption and the construction and demolition waste (CDW) in landfills [3]. Nonetheless, to reduce CO_2_ emissions and the carbon footprint of concrete, it is necessary to decrease the use of Portland cement (CEM I, 95% clinker) and increase the use of supplementary cementitious materials. In addition, the CO_2_ uptake capacity of cementitious materials must also be leveraged to enhance the ecoefficiency of concrete [4].

Recycled concrete aggregate (RCA), derived from the crushing of demolished concrete structures, contains adhered mortar that alters its properties relative to those of conventional aggregates. This residual mortar increases porosity, water absorption, and heterogeneity, which can adversely affect the workability, durability, and mechanical strength of concrete [5,6]. Among emerging solutions to enhance the quality of RCA, accelerated carbonation treatment has been established as one of the most promising techniques [7]. This process involves exposing RCAs to an atmosphere rich in carbon dioxide (CO_2_) under controlled conditions of temperature and humidity with the objective of inducing the transformation of hydrated cement compounds such as portlandite (Ca(OH)_2_) into calcium carbonate (CaCO_3_). Because carbonation treatment enhances the density of the adhered old mortar from the original concrete, the water absorption of carbonated RCA (cRCA) is less than that of the RCA and strengthens the interfacial transition zone, thereby improving the mechanical performance and durability of recycled aggregate concrete (RAC) [8,9]. Moreover, the carbonation process can capture 5–11 kg CO_2_/ton of RCA [7].

The fresh-state performance of concrete is affected by the high water absorption capacity of RCA, which causes a reduction in the flowability of self-compacting concrete (SCC) when RCA is incorporated into concrete [10,11]. This reduction is less pronounced when RAC contains cRCA [6].

Extensive research has demonstrated that with the appropriate processing, mix design, and curing strategy, concrete incorporating RCA can attain mechanical and durability performance comparable to that of conventional concrete—particularly when only moderate levels (20–60%) of coarse natural aggregate (NA) are replaced by RCA [12,13]. In addition, concrete incorporating cRCA can achieve a 15–20% increase in strength, depending on the type of aggregate incorporated and the carbonation conditions [14,15,16,17].

Furthermore, the use of RCAs in concrete production often results in increased drying shrinkage compared with concretes produced with NAs, primarily due to the higher water absorption capacity of RCA concretes [18]. In contrast, cRCA reduces the dry shrinkage of RAC—relative to concrete produced with uncarbonated RCA [6,19]. The shrinkage value is also dependent on various other factors, including compressive strength, environmental conditions, and the properties of cement and additions [20]. Early-stage shrinkage is particularly critical, as it contributes substantially to the final shrinkage magnitude and thus elevates the risk of cracking in the later stages of the concrete’s lifecycle [21,22]. Vintimilla and Etxeberria [23] found that structural concretes produced with up to 60% coarse RCA achieve shrinkage values similar to those of natural aggregate concrete (NAC).

Based on sorptivity and carbonation resistance—two indicators of durability—it has been demonstrated that RAC exhibits worse performance than conventional concrete when both are produced using the same cement type and effective water-to-cement (effective w/c) ratio [24,25]. Still, RAC achieves superior performance to conventional concrete if it is produced with a lower effective w/c ratio than the conventional concrete [23]. Incorporating cRCA into concrete further enhances the carbonation resistance of RAC, suggesting that the risk of steel reinforcement corrosion in concrete may be lower than in concrete made with uncarbonated RCA [26].

Regarding sorptivity, Russo and Llolini [27] explained that the RAC produced using 30% cRCA decreased by 8% compared to RAC made with 30% uncarbonated RCA. Dundar et al. [28] also reported that mortars produced with 100% of cRCA achieved a significant decrease in the sorptivity coefficient compared to that of the mortar made using uncarbonated RCA.

The carbonation curing method, also known as CO_2_ curing, is mainly suited for precast concrete because it requires a closed curing chamber for effective control of the process. Carbonation curing has primarily been studied using thin concrete specimens, which allow for rapid CO_2_ diffusion throughout the material [29]. The formation of a large number of calcium carbonate crystals on the outer surfaces of the concrete reduces the porosity of the surface zone, creating a barrier that further slows the rate of CO_2_ mineralisation [30]. Notably, several researchers [31,32] have studied the effect of early carbonation curing and concluded that performing the CO_2_ curing process at an early stage increases the surface resistance of concrete to chloride ion penetration, and increases the carbonation resistance of concrete. In addition, Sharma and Goyal [33] reported that carbonation curing concrete showed a reduction in surface permeability, resulting in decreased water absorption, sorptivity, chloride permeability, and carbonation depth. However, excessive carbonation can cause degradation of calcium silicate hydrate (C-S-H), the primary hydration product and binder in concrete, leading to a decrease in strength [29].

A pre-conditioning stage is crucial for effective carbonation curing, as it regulates the removal of excess water from the mixture after casting and before exposure to CO_2_. Excessive water content can hinder carbonation by blocking CO_2_ diffusion to reactive sites, while insufficient moisture content can result in incomplete reactions and poor carbonation efficiency [29]. In certain CO_2_ curing studies [31,34,35], a fan was used to dry concrete samples at a wind speed of 1 m/s prior to carbonation, aiming to remove 40% of the free water within 6–7 h and achieve carbon dioxide penetration and carbonate precipitation.

Due to the potential pH reduction caused by carbonation, which can lead to the corrosion of reinforcing steel in concrete [36], most studies on CO_2_-cured concrete [34,37,38,39] have employed relatively short CO_2_ curing durations, typically ranging from 2 to 24 h.

A significant portion of the available literature focuses on enhancing the mechanical properties of RAC through the carbonation process. However, fewer studies have explored its durability in detail. This research aims to contribute to a better understanding of RAC’s durability under carbonation.

This study analyses the impact of carbonated recycled aggregates and the CO_2_ curing process on improving the properties of structural SCC. The control mix proportion (conventional concrete produced with 100% NAs) was provided by a precast company based in Spain. The RCA used in this research was produced in the company’s precast plant by crushing and screening its own SCC waste. Currently, the company produces conventional concrete, which is made using 100% natural limestone aggregates and Portland cement (CEM I). In this research, an analysis was conducted on the use of RCA, low-carbon cement, and carbonation processes for aggregates, as well as curing processes, to elucidate an approach to producing highly sustainable concrete. RACs were produced using 50% and 60% coarse RCA (carbonated and uncarbonated) and two types of cement—ordinary Portland cement (CEM I) and cement CEM II B-M Portland composite cement containing 24% less clinker than CEM I—all with similar compressive strengths. After evaluating the CO_2_ curing process, the physical, mechanical, shrinkage, and durability properties (including suction and carbonation resistance) of the concretes were assessed. The RAC values were compared with those achieved with conventional concrete to generate insights for developing a highly sustainable concrete manufacturing process. 

## 2. Materials

### 2.1. Cement and Admixtures

Two types of cement were used in this study: CEM I 52.5 R Portland cement, with high initial strength and a 28-day compressive strength of 52.5 MPa, and containing 95% clinker; CEM II/B-M (P-L) 42.5 R Portland composite cement, with a high initial strength, a 28-day strength of 42.5 MPa, and 14% pozzolan and 10% limestone as the main constituents in replacement of clinker, both manufactured by Cementos Portland Valderrivas S.A., in Santa Margarida i Els Monjos, Barcelona, Spain. The chemical compositions of these cements are summarised in Table 1. X-ray fluorescence (XRF) was determined using a Wavelength-Dispersive X-ray Fluorescence Spectrometer, model PERFORM’X (Thermo Fisher, Waltham, MA, USA). The LOI temperature was set at 950 °C.

A high-performance water-reducing superplasticiser (SP), MasterEase 5025, manufactured by Master Builders Solutions Spain, S.L.U., in Cornellà de Llobregat, Barcelona, Spain, based on innovative phosphonic poly-aryl ether (PAE) polymers, which improve the rheological behaviour of concrete, was used to achieve the desired concrete workability.

### 2.2. Natural Aggregates (NAs)

Crushed limestone was used as the NA in the concrete mixes. Fine NA (FNA) with a particle size of 0/2 mm and coarse NA (CNA) with a particle size of 6/12 mm were used in the control concrete (CC) developed by HORMIPRESA NEC, S.L., in El Pla de Santa Maria, Tarragona, Spain, a precasting company.

**Figure 1:**
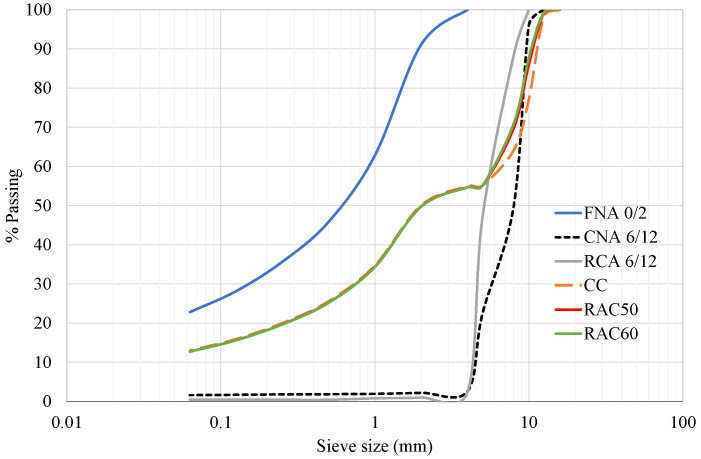
Particle size distribution of natural aggregates, recycled aggregates, and concretes.

The dry density and water absorption capacity of the aggregates were determined in accordance with the EN 1097-6 standard [40]. The values of these properties are presented in Table 2. The grading distribution was determined in accordance with the EN 933-1 [41] standard (sieving method) and validated following the EN 12620 specification [42]. The particle size distribution of the NAs (FNA 0/2 and CNA 6/12) are illustrated in Figure 1. The FNA had a high fines content (particles smaller than 0.063 mm), with 23% of the aggregate passing a 0.063 mm sieve, which increased the fines amount available for SCC production.

### 2.3. Recycled Concrete Aggregates (RCAs)

The RCA used in this study was sourced from concrete waste produced at a structural precast concrete plant. After crushing and sieving, the coarse fraction of the RCA was obtained for use in this study. The grading distribution (RCA 6/12), which was determined in accordance with EN 933-1 [41] and in compliance with the requirements of EN 12620 [42], is outlined in Figure 1. The RCA (6/12 mm) was finer than the CNA (6/12), with 57% and 22%, respectively, passing through an 8 mm sieve.

The key physical properties of the RCA—dry density and absorption capacity—are described in Table 2. Although the RCA had a higher absorption capacity than the CNA, in compliance with the requirements established by the Spanish Structural Concrete Code (SC-BOE) [43] the absorption capacity of the RCA was less than 7%.

### 2.4. Carbonated Recycled Concrete Aggregates (cRCAs)

Carbonation increases the density of attached cement paste and decreases the water absorption of RCAs [15]. The RCAs (6/12 mm) in this study were carbonated via subjection to a carbonation process using a carbonation chamber. To determine the most efficient carbonation process, two methods were compared for carbonation of the RCA. The first process involved exposing RAC to a 20% concentration of CO_2_ at 20 °C and 57% relative humidity for four days. The second method used the same CO_2_ concentration and relative humidity but with a higher temperature of 40 °C and a lower exposure time of two days. According to Wang et al. [44], high temperatures enhance the penetration rate of carbon dioxide and the substance exchange rate, thereby accelerating the carbonation process. It was observed that the conditions of the two carbonation methods yielded cRCAs with similar physical properties. For example, the absorption capacity of the cRCA carbonated at 20 °C for 4 days and that of the cRCA carbonated at 40 °C for 2 days was 5.65%. Consequently, the carbonation process was carried out at 40 °C for 2 days in order to minimise process duration.

The cRCAs achieved a slightly higher density and a 12.5% lower absorption capacity than the RCA. These results are consistent with the conclusion of previous some studies that carbonation can reduce the water absorption of RCAs by approximately 12–30% [8,45]. The extent of this reduction depends on the specific carbonation conditions and the properties of the RCA. In this study, the cRCA achieved an absorption capacity of 5.65%. This is a low absorption capacity for a recycled aggregate, considering that the water absorption capacity of recycled aggregates typically ranges from 3% to 12% [1], and for use in Spain, the water absorption capacity of RCAs is limited to 7% [43].

The abrasion resistance of the aggregates was evaluated using the Los Angeles abrasion test. The coefficient for the CNA was 28, while the RCA had a slightly higher coefficient of 30.

## 3. Concrete Production and Test Procedure

### 3.1. Concrete Production

Two series of concretes were produced: Series 1, using CEM I (with a 52.5 strength class); and Series 2, using CEM II/B (with a 42.5 strength class). In addition, to achieve similar compressive strength in both series concretes, the Series 1 concretes were designed with an effective w/c ratio of 0.49 and a cement content of 340 kg/m^3^, while the Series 2 concretes were produced with an effective w/c ratio of 0.45 and a cement content of 365 kg/m^3^.

The Series 1 CC mixture (CC-I) was provided by a Spanish precast concrete company. The concrete mixes were designed for exposure class XC (carbonation-induced corrosion) per the UNE-EN 206 standard [46].

Five concrete mixes were produced in each series (Table 3): CCs produced with 100% NA; RAC50 and RAC60 concretes, produced using 50% and 60% RCA, respectively; and RAC-C50 and RAC-C60, produced using 50% and 60% cRCA, respectively, as replacements for CNA.

The total particle size distribution of the aggregates employed in producing the concretes (CC, RAC50, and RAC60) is outlined in Figure 1. The grading distribution exhibited a discontinuity between the 2 mm and 6 mm size range, corresponding to the transition between fine and coarse aggregates, which could compromise particle size continuity and may negatively impact the workability and compactness of the concrete. Among the RACs, the CC achieved a superior overall particle size distribution, owing to finer coarse RCA particles compared with those in the CNA mix. Notably, a gap in the grading between coarse and fine aggregates is used in some mix designs, in accordance with the European Guidelines for SCC [47].

The concrete mixtures used in this study were designed to achieve the SF2 classification (660–750 mm) in a slump flow test, indicating adequate consistency and flowability. Viscosity was evaluated simultaneously by determining the t_500_ time during the slump flow test, which was ≥2.0 s—corresponded to class VS2. Both tests were conducted in accordance with the EN 12350-8 specification [48].

The Series 1 and Series 2 concrete mixtures are denoted by the I and IIB symbols, respectively, highlighting the cement type in the mix designation (Table 3). The moisture content of the aggregates was determined before producing each batch of concrete to accurately control the effective w/c ratio and fresh-state properties. The humidity of the recycled aggregates was maintained at 70–80% of their absorption capacity [12].

**Table 3:** Mix proportions of Series 1 and Series 2 concretes.

Mix	Cement (kg)	Total Water (kg)	NFA (kg)	NCA (kg)	RCA (kg)	CRCA (kg)	SP (%)	W/C ef.	Slump Flow (mm)	Viscosity t_500_ (s)
Series 1										
CC-I	340	185.1	1055	891			2.5	0.49	730	5
RAC50-I	340	208.7	1055	446	389		2.5	0.49	760	3
RAC60-I	340	214.8	1055	356	466		2.5	0.49	750	4
RAC-C50-I	340	205.3	1055	446		394	2.5	0.49	730	5
RAC-C60-I	340	209.9	1055	356		476	2.5	0.49	750	4
Series 2										
CC-IIB	365	184.8	1046	884			2.6	0.45	720	6
RAC50-IIB	365	206.4	1046	442	385		2.6	0.45	690	8
RAC60-IIB	365	210.0	1046	354	463		2.6	0.45	720	8
RAC-C50-IIB	365	202.9	1046	442		390	2.6	0.45	720	6
RAC-C60-IIB	365	205.9	1046	354		473	2.3	0.45	730	9
EN-12350-8 [48]									660–750	≥2.0 s

The concretes were produced using a vertical shaft mixer with a 60-litre capacity. For the mixing process, coarse and fine aggregates were first mixed with a little water for 20 s. After which cement was then added to the mixer, and the remaining water was added gradually, followed by the SP. The amount of water added to the mixer was the effective water (the amount water defined by the effective w/c ratio) plus the water absorbed by the aggregates. Finally, the mixture was mixed for another minute to ensure that all the components were thoroughly and homogeneously mixed before testing the fresh-state properties.

After mixing, the fresh-state properties (slump flow and t_500_ time) were determined for all SCC mixtures produced. In Table 3, it can be seen that all the concretes produced—except for RAC50-I—achieved the required slump flow value at a diameter of 660–750 mm, corresponding to the SF2 classification. RAC50-I was also produced with 2.5% (by weight of cement) of SP; however, due to the lower humidity of the RCA, a comparatively large amount of water was added to the mixer to be absorbed by the RCA, resulting in a diameter larger than the upper limit of the SF2 classification. In addition, the flow time to achieve a spread diameter of 500 mm was satisfactory for all Series 1 and Series 2 concretes.

The Series 1 and Series 2 concretes required approximately 2.5–2.6% of SP (relative to the weight of the cement) to achieve the required slump flow and t_500_ time values (Table 3). In addition, contrary to expectations, the amount of SP remained consistent across all mixtures, and increasing the dosage was not necessary when 50–60% coarse NA was replaced by RCA or cRCA. Safiuddin et al. [49] examined various levels of substitution of CNA by RCA and found that the fresh-state properties of SCC produced with up to 50% coarse RCA in replacement of CNA were similar to those of conventional concrete.

The concrete type of the specimens used in testing the hardened concrete properties for each concrete mixture is listed in Table 4; because of the properties of SCC, no compaction was required. All the concrete specimens underwent steam curing (S-process), and half of the specimens were then additionally subjected to CO_2_ carbonation—thus undergoing a steam carbonation process (S-C process)—to evaluate the efficiency of these processes in improving the durability properties of concretes.

### 3.2. Curing Processes

In this study, the production process of the precast concrete factory was maintained. The concrete precast element was required to have a compressive strength of 25 MPa before demoulding. Consequently, to achieve a high initial strength, the concrete specimens were cured in a steam chamber at a temperature of 38 ± 2 °C and a relative humidity of 75 ± 5% for 8 h (i.e., the S-process). After the S-process, the specimens were demoulded, and their initial weight was recorded to monitor water loss. In addition, the compressive strength of all the concretes produced was determined to verify the required minimum strength (25 MPa). Afterwards, the specimens were stored on a shelf in a laboratory room under atmospheric environmental conditions, with a temperature of 19 ± 2 °C and a relative humidity (RH) of 51 ± 6%. This storage method is similar to that used by the precasting company, exposing the specimens to atmospheric environmental conditions before testing or placing them on-site.

Furthermore, to evaluate the variability in the durability properties of the concrete, half of the concrete specimens—which had undergone the S-process and then been exposed to atmospheric environmental conditions (temperature: 19 ± 2 °C, humidity: 51 ± 6%) for two days (precondition duration)—were additionally subjected to a CO_2_ curing process, thus undergoing the S-C process. The precondition duration of two days was determined by achieving a moisture loss equivalent to approximately 30% of their absorption capacity, being approximately 20% of water mass loss in CCs, a similar value to that determined by Han et al. [55]. Reducing the moisture content of the concrete specimens was required to facilitate CO_2_ penetration and promote calcium carbonate precipitation [31,34,35]. To determine the optimal duration for the CO_2_ curing process—during which the specimens were exposed to an atmosphere containing 20% CO_2_, a temperature of 20 °C, and 57% RH—two variants of the CO_2_ curing process were performed: Duration 1, performed for a duration of 24 h; and Duration 2, lasting 48 h.

#### Optimisation of the CO_2_ Curing Process: Duration 1 and Duration 2

CO_2_ curing of concrete is performed to make the surface layer of the concrete stronger and more durable. The formation of carbonate deposits during this process seems to produce the structure of the concrete denser and less porous [31]. As a result, the concrete becomes less vulnerable to penetration by harmful ions in the environment.

To evaluate whether 24 or 48 h is the optimal duration for the CO_2_ curing process (20 °C, 20% CO_2_, 57% RH), the capillary water absorption coefficient (per the UNE-EN-ISO-15148 specification [53]) and the carbonation depth (per the UNE-EN 14630 specification [56]) were determined in 100 × 100 × 100 mm cubic specimens of the CC and RAC-C50 concretes. Following subjection to the CO_2_ curing process, the specimens were stored under laboratory conditions (20 °C and 50% RH) until the test date—at an age of 15 ± 2 days.

The results (Table 5) show that increasing the CO_2_ curing time decreases the sorptivity (capillary absorption coefficient) of the concrete and increases the carbonation depth. On average, the carbonation depth exceeded 2 mm after one day of curing and surpassed 4 mm after two days. Based on these findings—considering that the initial carbonation depth of 2 mm could be sufficient for the formation of a dense surface layer that effectively reduces the penetration of harmful agents into the interior of the concrete’s—the CO_2_ curing process with a one-day duration was selected for the S-C process.

After CO_2_ curing, the specimens were again stored under environmental conditions of 19 ± 2 °C and 51 ± 6% RH until the testing day. The results obtained for the concrete specimens that underwent only the S-process and those that underwent the S-C process were assessed to analyse the CO_2_ curing effect.

## 4. Test Procedure

Table 4 summarises the tests performed—and the standards followed—to assess the hardened concrete properties of the concrete specimens produced, as well as the test age and the concrete type of the specimens used. After undergoing the S-process for 8 h, the samples were demoulded and stored on a shelf until they reach an age of 24 h. The compressive strength at both ages (8 h and 24 h) was determined using two and four specimens, respectively. In addition, the specimens were tested for compressive strength at 7 and 28 days using two and four specimens, respectively, after being subjected to the S-process or the S-C process and stored in laboratory conditions of 19 ± 2 °C and 51 ± 6% RH until the time of testing. Furthermore, density, absorption, and sorptivity were tested at an age of 28 days after undergoing either of the two curing methods (S or S-C), using two cubic specimens for each property.

The capillary water absorption and sorptivity were evaluated using cubic specimens measuring 100 × 100 × 100 mm at an age of 28 days, in accordance with ISO 15148:2002(E) specifications [53]—after being conditioned in an oven at 40 °C for 72 h. To conduct the test, the bottom faces of the specimens were submerged in 5 mm of water, and the lateral surfaces were treated with an impermeable resin. The cumulative amount of water absorbed by each specimen was recorded at various time intervals over a period of 24 h. Sorptivity was determined as the slope of the regression curve plotting the quantity of water absorbed per unit surface area against the square root of the elapsed time from the initial moment (t = 0 min) to 120 min [5]. The recorded results represent the average of the two measurements.

To determine carbonation resistance, the concrete specimens, after being subjected to the two curing methods (S or S-C) and being exposed to laboratory conditions of 19 ± 2 °C, 51 ± 6% RH, and 430 ppm of CO_2_ for 28 days—the average duration for which concrete is air cured under atmospheric conditions by the precasting company—the cement specimens were kept in a CO_2_ chamber at 20 °C in an atmosphere containing 3% CO_2_ and 57% RH for 70 days. The carbonation depth of the two concrete specimens for each concrete type and curing procedure was determined at 7, 28, 56, and 70 days after exposure to CO_2_. The carbonation depth was determined using a solution of 1 g phenolphthalein in 70 g ethanol and 30 g water, in accordance with UNE-EN 14630 [56].

Following the procedure for the drying shrinkage test, once the concrete specimens reached ambient temperatures after the S-process, their initial length and mass were measured. Subsequently, the length and mass of all specimens were measured on Day 1 (all the concrete specimens were subjected to the S-process) and on Day 3, 5, 7, 14, 21, 28, and 56 after casting; half of the specimens were subjected to the S-process and half to the S-C process. The shrinkage strain values and mass losses of each concrete type were determined. Two concrete specimens for each concrete type and curing procedure were used.

## 5. Results

### 5.1. Physical Properties

Table 6 presents the data on the following physical properties achieved by all the concrete specimens at an age of 28 days: dry density, absorption, and accessible porosity.

In terms of density, RCA concretes exhibit a slight decrease—3% and 2% for Series 1 and Series 2, respectively—compared to CCs. Furthermore, the curing process did not result in any significant variation in this property.

Regarding absorption capacity, the incorporation of RCA led to an increase in RACs compared to CCs. This finding is consistent with Xuan et al. [18], who explained that replacing natural aggregates (NAs) with recycled concrete aggregates (RCAs) increases the water absorption of RAC due to the inherently higher absorption capacity of RCAs. The most significant increases were recorded for the RCA-C50-I and RCA50-IIB concretes, showing 32% and 18% increases in Series 1 and Series 2, respectively. These concretes obtained the lowest slump flow value and consequently the lowest compaction capacity.

Some researchers have found that cRCAs exhibit a lower water absorption capacity than RCAs, owing to the carbonation and densification that RCAs undergo to produce cRCAs, which leads to a low water absorption capacity in RACs [18]. However, this study did not reveal a general trend. Among the Series 1 concretes, RAC-C60-I achieved a lower absorption capacity than RAC60-I, while RAC-C50-I achieved a higher absorption capacity than RAC50-I. In contrast, among the Series 2 concretes, RAC-C50-IIB achieved a lower absorption capacity than RAC50-IIB, while RAC-C60-IIB achieved a higher absorption value than RAC-60-IIB. Neville A.M. [5] posits that absorption capacity cannot be used as a definitive measure of concrete quality; however, good-quality concretes typically have an absorption capacity of well below 10% by mass. Furthermore, Cantero et al. [57] report that an effective porosity of <15% is indicative of high-quality concrete. Based on the test results in this study, all the RACs produced were considered acceptable. Comparing the Series 1 and Series 2 concretes—with respect to the impact of the cement type used—it was observed that concretes produced with IIB cement achieved a lower density and higher porosity and absorption capacity than those produced with type I cement (Table 7).

**Figure 2:**
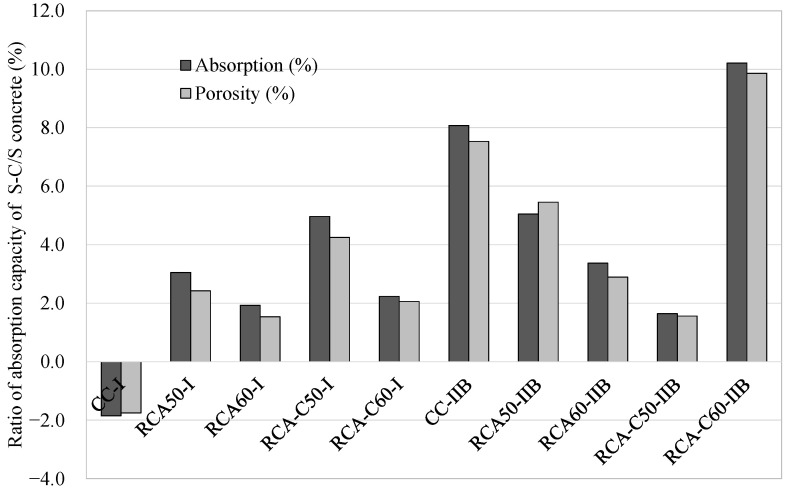
Influence of CO_2_ curing on the porosity absorption capacity of concretes.

The additional CO_2_ curing improved the absorption and porosity of all the concretes produced using the S-C process compared with those produced using only the S-process (steam curing). Figure 2 captures the relationship between porosity and absorption capacity in CO_2_-cured concrete (i.e., concrete that underwent the S-C process) in comparison to the equivalent specimens that did not undergo CO_2_ curing (i.e., concrete that underwent only the S-process). The concrete specimens subjected to the CO_2_ curing of the S-C process exhibited a slightly enhanced physical properties compared with those that did not undergo the S-C process—except for CC-I, which was subjected to the S-C process but registered only a 2% higher absorption capacity than corresponding samples that underwent only the S-process. The greatest reductions in absorption and porosity were observed in RAC-C60-IIB, with a 10% decrease in both properties. Following RAC-C50-I and RAC-50IIB, which showed absorption reductions of 5%, and porosity decreases of 5.5% and 4.2%, respectively. However, it should be noted that the CO_2_ curing caused surface carbonation (2 mm depth), which had a minimal impact on the physical properties of the concrete. This effect was greater in the concretes produced using CEM II/B than those produced using CEM I.

### 5.2. Compressive Strength

The concrete specimens were demoulded after undergoing the S-process and stored under laboratory conditions (19 ± 2 °C and 51 ± 6% RH) before testing. The compressive strength was determined using 100 × 100 × 100 mm cubic specimens at 8 h (after casting and demoulding) and 24 h after casting. In addition, the specimens subjected to the two curing methods, the S-process and the S-C process (Table 7), were tested at 7 days and 28 days after casting.

It should be noted that initial steam curing (S-process) can lead to an increase in strength at early ages by accelerating chemical hydration reactions [58]. However, initial steam curing may not be entirely beneficial, and strength may decrease after 28 days [59]. Hence, some of the concrete specimens were exposed to laboratory conditions instead of standard curing conditions (i.e., a humidity room) after undergoing the steam curing and CO_2_ curing (S-C process). Consequently, these specimens lost water, which impacted their strength development compared with conventional wet curing.

In Table 7, it can be seen that after 8 h of the S-process, all the concrete specimens exceeded the compressive strength of 25 MPa set as the threshold for demoulding by the precast company. At 24 h (1 d) after casting (i.e., being subjected to the S-process and kept under laboratory conditions until reaching the testing age), all the concrete specimens exhibited an increase in compressive strength of over 20% compared with their compressive strength at an age of 8 h—except for RAC-C60-IIB. Notably, the CC-I and C-IIB concretes exhibited the largest gains, with an increase of 27%; in contrast, the compressive strength of RAC-60-IIB at an age of 1 day had increased by 11% with respect to its compressive strength at 8 h, since at 8 h it achieved the highest strength.

At an age of 7 days, the compressive strength of the concrete specimens had increased significantly compared with the value at an age of 1 day. For both Series 1 and Series 2 concretes, there was an average increase of approximately 47% when the concrete was produced using only the S-process. For concretes subjected to the S-C process, the average increase in strength from Day 1 to Day 7 was 52%. These concrete specimens, after being subjected to the CO_2_ curing process, achieved higher compressive strength on Day 7 (Table 7). Furthermore, compressive strength increased from Day 7 to Day 28 by an average of approximately 15% for Series 1 concretes, and 17.5% for Series 2 concretes. However, the concretes produced using the S-C process achieved a 5% lower increase in compressive strength from Day 7 to Day 28 compared with those produced using only the S-process, which achieved a 15% increase. Regardless of S-process or S-C process, on average, the 7-day compressive strength reached 86% of the 28-day value.

Based on the results recorded for the concrete specimens at 28 days of age, all the concretes exhibited similar and high compressive strengths of up to 62 MPa. In addition, it was observed that the mix designs of Series 1 and Series 2 concretes—which used different cement types, with effective w/c ratios of 0.49 and 0.45, respectively—achieved similar strengths.

**Figure 3:**
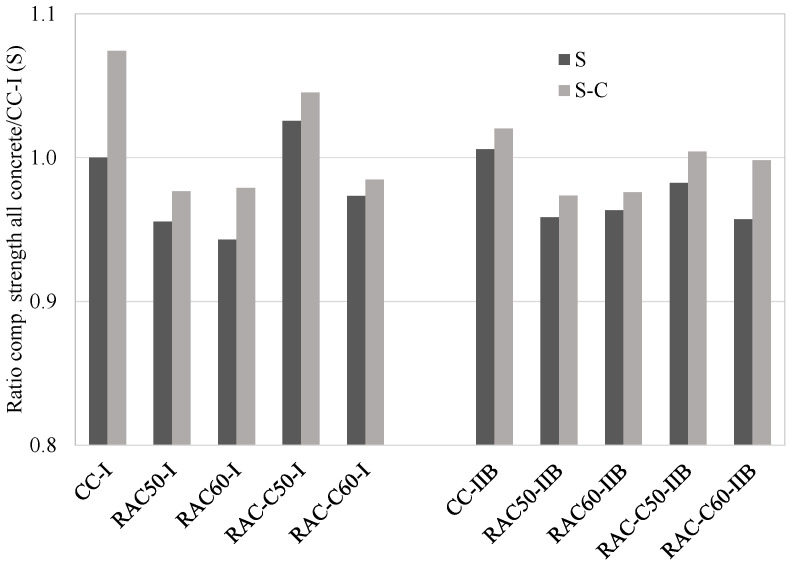
Compressive strength ratios at 28 days for all concretes produced, with respect to that of CC-I (S-process).

Figure 3 presents the compressive strength ratios of each concrete produced with respect to that of CC-I (S-process CC from the precast company). It was observed that all the concrete specimens produced using the S-C process achieved a slight increase in compressive strength as a result of the CO_2_ curing, compared with the concrete specimens produced using the S-process alone. In addition, it can be seen in Figure 3 that RAC-C50-I produced using the S-process or S-C process achieved an increase in compressive strength of 2.6% and 4.5%, respectively—higher compressive strengths than CC-I (S-process). Regarding RAC, among the Series 1 concretes, RAC60-I (S-process) achieved the lowest strength at 28 days, 6% lower than that of CC-I (S-process). Furthermore, RAC60-I (S-C process) progressed efficiently and achieved only a 2% lower strength than CC-I (S-process). Among the Series 2 concretes, RAC-C50-IIB and RAC-C60-IIB, which were produced using the S-C process achieved a compressive strength similar to that of CC-I and CC-IIB, which were produced using the S-process. RAC-C60-IIB, after being subjected to the S-C process, achieved the highest increase with respect to that of the S-process, increasing by almost 5%.

### 5.3. Drying Shrinkage

**Figure 4:**
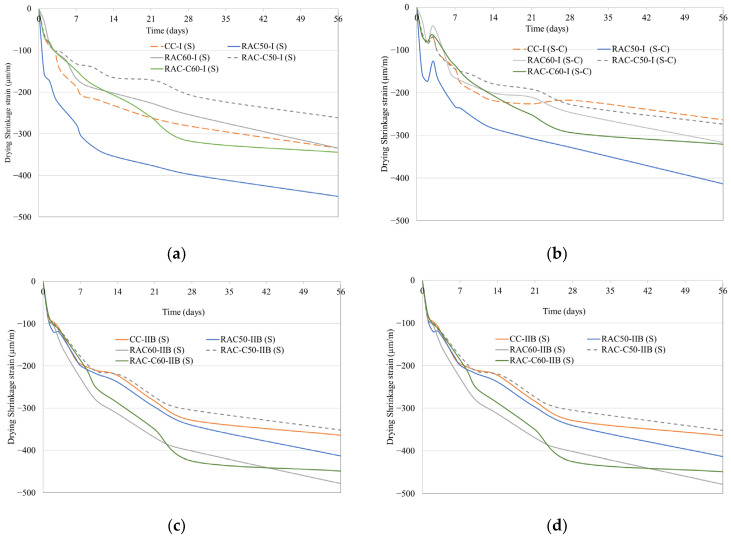
Drying shrinkage of concretes: (**a**) CEM I and steam curing (S-process); (**b**) CEM II/B, steam curing and CO_2_ curing (S-C process); (**c**) CEM II/B and steam curing (S-process); and (**d**) CEM II/B, steam curing and CO_2_ curing (S-C process).

Figure 4 illustrates the drying shrinkage (µƐ) of all concretes produced for up to Day 56. In addition, the shrinkage value and mass loss for all the concrete specimens after 56 days stored on a shelf in a laboratory room under atmospheric environmental conditions are presented in Table 8. Figure 4a,b provide the shrinkage values for Series 1 concretes produced using the S-process and the S-C process, respectively. In addition, Figure 4c,d provide the shrinkage values for Series 2 concretes produced using the S-process and the S-C process, respectively. All the concrete specimens exhibited a similar trend: the most significant shrinkage occurred at early ages, followed by stabilisation over time. For Series 1 concretes, when the samples were cured using the S-process (Figure 4a), RAC-C50-I exhibited the lowest shrinkage value (−262 με), followed by CC-I, RAC-C60-I, and RAC-60-I, which all recorded values lower than 350 με (Table 8). RAC-C50-I exhibited 22% less shrinkage than CC-I. In contrast, RAC-50-I achieved the highest shrinkage value, probably due to a higher free water content at the moment of casting than the other Series 1 specimens, as indicated by the values from the flow slump test (Table 3). The concretes subjected to the S-C process achieved lower shrinkage values than those subjected only to the S-process (Figure 4b). Tam et al. [30] determined that CO_2_ injection into concrete has a significant impact on drying shrinkage. At 3 days (after the S-C process), the shrinkage values recorded for the specimens were reduced compared to the corresponding specimen subjected to the S process (Figure 4b), probably due to a higher RH in the CO_2_ chamber than in the laboratory, as well water released due to carbonation reaction [60].

After being subjected to the S-C process, a CC-I specimen achieved a 21% lower shrinkage than that subjected to the S-process, a shrinkage of −264 με. In addition, all the RACs produced with cRCA achieved a lower value than those produced with uncarbonated RCA. Similarly, the concretes produced using the S-C process achieved a lower mass loss than those produced using the S-process due to the high humidity in the CO_2_ chamber. All the concretes lost less than 3% of water mass, with CC-I recording the lowest value.

The shrinkage values of the Series 2 concretes produced using CEM II/B are presented in Figure 4c (S-process) and Figure 4d (S-C process). The Series 2 concretes produced using CEM II/B (14% of pozzolans and 10% limestone) achieved up to 40% higher shrinkage values than the corresponding concretes produced in Series 1 using CEM I. The use of supplementary cementitious materials (SCMs) increases the shrinkage of concretes [21]. In Figure 4c, it can be seen that RAC-C50-IIB and CC-IIB achieved the lowest shrinkage in their category (≈−350 µƐ), followed by RAC50-IIB (Table 8). RAC-C60-IIB and RAC-60-IIB achieved the highest shrinkage values of −449 µƐ and −478 µƐ, respectively. The use of RAC in concrete production reduces stiffness caused by the amount of adhered mortar in recycled aggregates [23,61,62]. In general, cRCA, due to its absorption capacity being lower than that of uncarbonated RCA (Table 3), reduces concrete shrinkage. Furthermore, the concretes subjected to the S-C process achieved up to 10% lower shrinkage values than those of concrete cured in the S-process, except for RAC-C50. CC-IIB achieved the lowest shrinkage value after undergoing the S-C process, followed by the concretes produced with 50% RCA. RAC60-IIB and RAC-C60-IIB achieved the highest shrinkage value of −443 µƐ. As mentioned above, the CO_2_ injection into concrete has a significant impact on drying shrinkage [30,45]. In addition, it also had the maximum mass loss with 2.9%.

**Figure 5:**
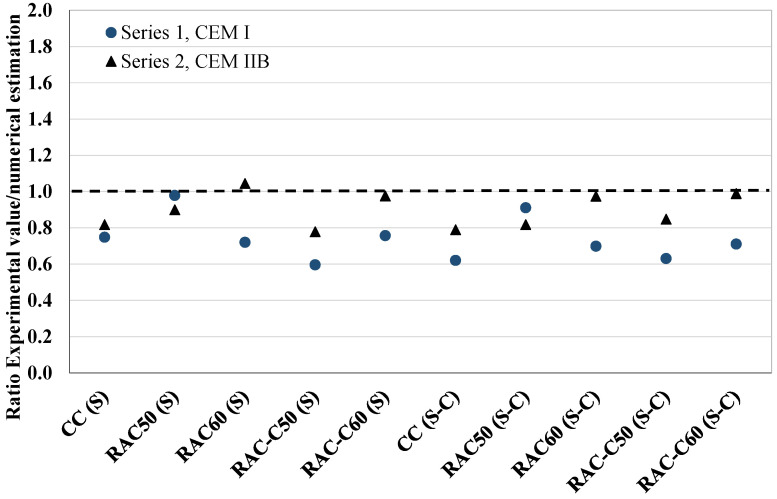
Analysis of shrinkage estimation, the ratio of experimental results/numerical estimation following EC-02 for the concretes produced in Series 1 and 2.

Under the given test conditions, a comparison of shrinkage behaviour among concrete specimens produced with different cement types revealed that IIB cement resulted in inferior performance to I cement. Notwithstanding the observed variations, the shrinkage values of all the concrete specimen produced were within the typical long-term shrinkage range mentioned above. All the concretes achieved low shrinkage values—long-term concrete shrinkage values should be between 200 × 10^−6^ and 800 × 10^−6^ mm/mm (200–800 µƐ) [63]. In addition, Figure 5 describes the ratio between the experimental values and the theoretical values based on Eurocode 2: EN 1992-1-1 [64]. The drying shrinkage is numerically estimated with Equation (1)

(1) $$\varepsilon_{cds(t-ts)}=\left[\left(220+210.\alpha_{ds}\right)exp\left(-0.012f_{cm,28}\right)\right].10^{-6}.1.55\left[1-{\left(\frac{RH}{99.{\left(\frac{35}{f_{cm,28}}\right)}^{3}\leq 99}\right)}^{3}\right].{\left[\frac{\left(t-ts\right)}{0.035*{hn}^{2}+\left(t-ts\right)}\right]}^{0.5}n_{shRA}$$

where α_ds_ is a coefficient that depends on the type of cement, which is 6 for R class cement; $f_{cm,28}$ is the compressive strength at 28 days in N/mm^2^; RH is the relative humidity of the environment; *t_s_* is the age of the concrete at the beginning of drying (1 day); *t* is the time elapsed since concrete casting (i.e., concrete age, which was 56 days); *h_n_*: is the notion size = 2 A_c_/u, where A_c_ is the concrete cross-sectional area and u is the perimeter exposed to drying; *η_shRA_* is a factor when RCA is employed in concrete production between 0.20 < α_RA_ ≤ 0.40 (*η_shRA_* = 1 + 0.8 α_RA_, where α_RA_ is a ratio between the quantity of RCA and the total quantity of aggregates, fine and coarse, employed). However, due to the adequacy of shrinkage values, probably resulting from steam curing [65] and CO_2_ curing, even in Series 2 concretes, the *η_shRA_* factor was not considered in this case. All the RAC, even the series 2 concretes, and more clearly the concretes submitted to the CO_2_ curing process, experimentally obtained lower shrinkage values than those estimated based on EC-2.

### 5.4. Durability Properties

Because the concrete specimens produced in this study must meet the quality requirements for exposure class XC4—environments in which there is a risk of carbonation-induced corrosion due to alternating cycles of moisture and drying—their performance, as indicated by their capillary water absorption coefficient (UNE-EN ISO 15148 [53]) and carbonation resistance assessed using the accelerated carbonation method (UNE-EN 12390-12 [54]) were determined.

#### 5.4.1. Capillary Water Absorption Coefficient—Sorptivity

**Figure 6:**
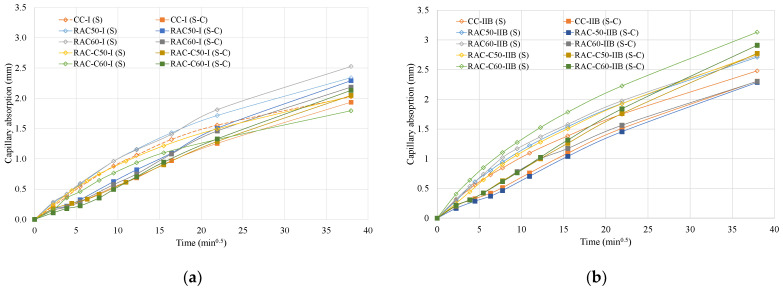
Capillary absorption values at 28 days for (**a**) Series 1 and (**b**) Series 2.

Table 6 presents the sorptivity values achieved by the concrete specimens produced in this study. Series 1 concretes produced using the S-process and made with RCA exhibited higher capillary absorption than those made with NA, except for RAC-C60-I, which had a lower capillary absorption value than CC-I. This observation is consistent with the findings of Aragoncillo et al. [66], who reported that water sorptivity, in general, tends to increase with the incorporation of higher volumes of RCA in the mix. In addition, the concrete specimens produced with cRCA exhibited a lower sorptivity value compared with their uncarbonated counterparts. RCA-C50-I and RCA-C60-I achieved 6% and 17% lower sorptivity values, respectively, than the corresponding concretes produced using uncarbonated RCA. Similar results were obtained by Russo and Llolini [27]. Furthermore, CO_2_ curing (S-C process) resulted in significant reductions in sorptivity values, ranging from 30% to 40%, compared with concretes that underwent only the S-process. In this context, concretes incorporating cRCA exhibited a lower sorptivity than those containing uncarbonated RCA, and even outperformed CC-I. Notably, RAC-C60-I achieved the lowest sorptivity value among the Series 1 concretes, measured at 0.052 mm/min^0.5^. Figure 6a presents the capillary absorption of the Series 1 concretes. During the first 4 h, the capillary absorption of the S-C concretes was lower than that of the concretes produced using only the S-process. However, over a more extended period, the absorption capacity values for RAC50-I and RAC60-I (S-C) increased to a greater degree than those for RAC-C50-I and RAC-C60-I, which remained low; RAC50-I and RAC60-I also achieved an adequate absorption capacity values.

In Series 2, all the concrete specimens subjected to the S-process achieved a high sorptivity value (Table 3), exceeding the sorptivity threshold of 0.10 mm/min^0.5^—considered the upper limit for durable concretes based on criteria established by Alexander et al. [67]. The RACs achieved a higher value than CC-II, and incorporating cRCA did not yield improvements in capillary absorption at the 60% substitution level. However, the application of CO_2_ curing (S-C process) resulted in a 25–45% reduction in the value of the sorptivity coefficient across all concretes—similar to the trend observed in Series 1. This reduction saw the coefficients falling below the 0.10 mm/min^0.5^ threshold, indicating enhanced durability performance. the values for the capillary absorption capacity of the Series 2 concretes are presented in Figure 6b. Similarly to Series 1 concretes, the Series 2 S-C concretes achieved a low absorption capacity in the first four hours; however, this increased after 24 h. However, the CC-II, RAC60-II, and RAC50-II, which were cured using the S-C process, had the lowest capillary absorption capacity values. This is confirmed by [68], who reported that CO_2_ curing at normal room temperature had a beneficial effect in reducing the water sorptivity of cement blocks.

Considering cement type (Series 1 vs. Series 2), the concretes produced with CEM II/B (Series 2) exhibited higher capillary absorption coefficients compared with those produced with CEM I. However, when subjected to CO_2_ curing, the performance of the CEM II/B concretes improved significantly, reaching levels considered acceptable for durable concrete.

#### 5.4.2. Carbonation Resistance

**Figure 7:**
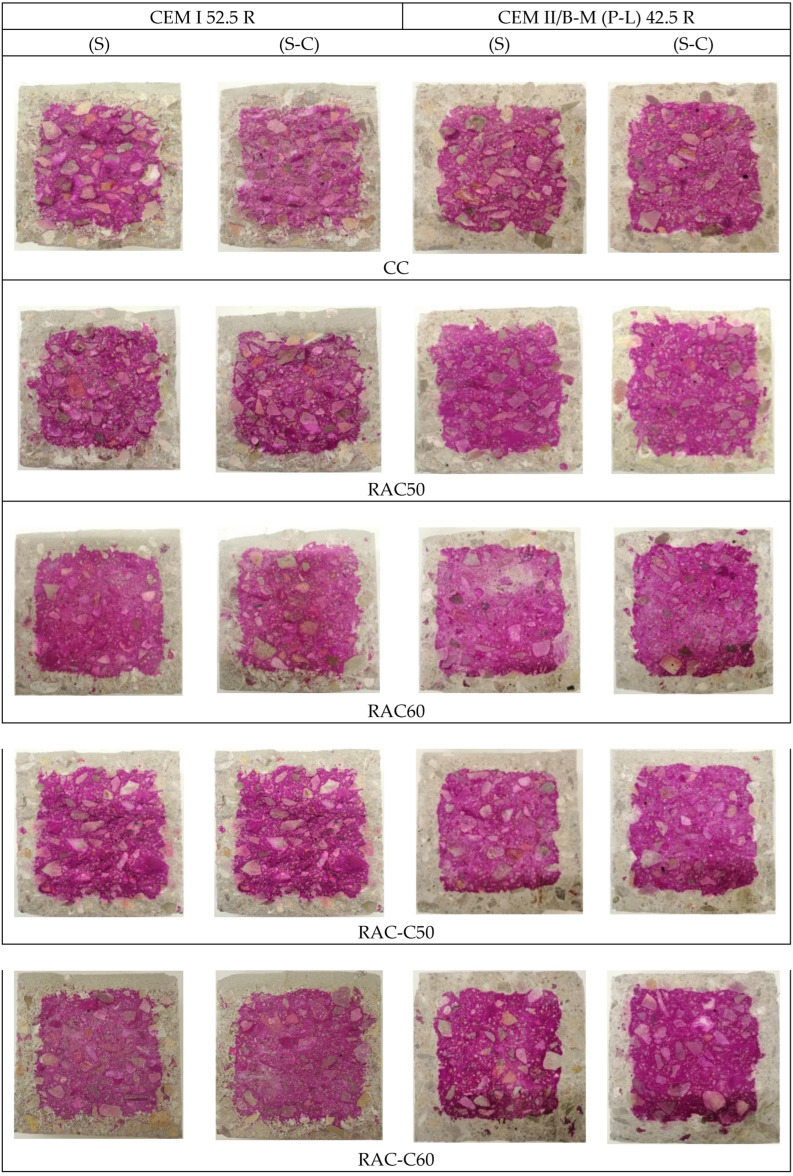
Visual comparison of the carbonation depth in accelerated conditions (70 days) for concretes.

The average carbonation depth after 70 days (subjected to 20 °C, 3% of CO_2_, and 57% RH) of Series 1 and Series 2 concretes and their accelerated carbonation coefficient (*Kacc*) are presented in Table 9. Figure 7 visually compares the carbonation depth obtained in the produced concretes under accelerated conditions over 70 days.

The *Kacc* value of each concrete was calculated under steady-state conditions based on Fick’s first law of diffusion, expressed as Equation (2)

(2) $$Xc(t)=Kacc\cdot {\left(t\right)}^{0.5}$$

where *Xc* is the determined carbonation depth (mm), *Kacc* is the accelerated carbonation coefficient (mm/day^0.5^), and *t* is time (days). The carbonation depth was determined at 0, 7, 28, 56, and 70 days of exposure to 3% CO_2_, 57% RH, and 20 °C.

In Series 1, the initial carbonation depth (on Day 0, when the specimens were placed in the CO_2_ chamber) was approximately 2 mm for all concretes subjected to the S-C process. In contrast, the concretes subjected to the S-process alone had an initial carbonation depth of approximately 0 mm. At 70 days of exposure to CO_2_ (Table 9), the carbonation depth of Series 1 RACs was lower than that of the CC-I. CC-I, which underwent only the S-process, achieved the highest carbonation depth. In addition, the concrete produced with cRCA achieved the lowest carbonation depth. RAC-C50-I had the lowest *Kacc* value of all the concrete specimens produced, at 1.28 mm/day^0.5^. However, the carbonation resistance of all the concretes produced using the S-C process was greater than those produced using only the S-process by 16–22%. As mentioned earlier, CC-I subjected to the S-process achieved the highest *Kacc* value, 1.34 mm/day^0.5^; however, undergoing CO_2_ curing (S-C process) decreased the *Kacc* value recorded after the S-process by 18%. RAC-C50-I achieved the lowest carbonation rate, followed by RAC-C60-I, RAC50-1, RAC60-I, and CC-1. Liu and Meng [32] described that carbonation-cured cement-based materials are less vulnerable to atmospheric CO_2_ than normally hydrated ones.

Among the Series 2 concretes produced using CEM II and an effective w/c ratio of 0.45, the initial carbonation depth (Day 0) of the concrete specimens that underwent the S-C process was approximately 2.6 mm. That is, except for RAC50 and RAC60, which achieved initial carbonation depths of 1.8 mm—a slightly higher value than that achieved by the Series 1 concretes. This is probably due to the use of Type IIB cement, which had 24% less clinker than CEM I [60]. The use of pozzolans resulted in a reduction in the total alkaline content that can be carbonated, leading to greater carbonation depths [69]. The Portlandite is reduced, and its carbonation reduces pore size and meso- and macropore volume due to CaCO_3_ precipitation [60], decreasing carbonation rate. In addition, the supply of Ca(OH)_2_ from uncarbonated RCA increased the carbonation resistance of the Series 1 concretes [70], resulting in lower initial carbonation depths than those achieved by the Series 2 concretes. After 70 days, the carbonation test results for the Series 2 concretes showed that the CCs, RAC50, and RAC60 achieved lower carbonation depths than the Series 1 specimens due to a lower effective w/c ratio than that used for the Series 1 specimens. However, RAC-C50 and RAC-C60 (produced in Series 2) achieved higher carbonation depths than Series 1 specimens due to the influence of high porosity and low calcium hydroxide in carbonated aggregates, which reduces carbonation resistance [70]. RAC-C60, subjected to the S-process, achieved the highest carbonation rate. Similarly to Series 1, all the concretes produced using the S-C process achieved a lower carbonation rate than the corresponding concrete specimens produced using the S-process. RAC50 (S-C process) achieved the lowest carbonation rate (1.14 mm/day^0.5^)**,** followed by RAC60, RAC-C60, CC, and RAC-C50.

Based on the Spanish Structural Code [43], in order to have a life span of 50 years, concretes exposed to a class XC4 environment, with a compressive strength of 25–40 MPa, must be built with a minimum concrete cover of 20 mm when CEM I is used and 25 mm when CEM II/B is employed. In addition, concretes with a 100-year lifespan need to have a minimum concrete cover of 30 mm when CEM I is used and 35 mm when CEM II/B is employed.

The *Kacc* value can be used to estimate the theoretical natural carbonation coefficient (*knatTHEO*) [71,72] of each type of concrete produced (Table 10) using Equation (3).

(3) $$\frac{Kacc}{KnatTHEO}=\frac{{\left(\emptyset acc\right)}^{0.5}}{{\left(\emptyset natTHEO\right)}^{0.5}}$$

where ∅acc and ∅natTHEO are the CO_2_ concentrations used in the accelerated carbonation (3%) and natural carbonation processes (430 ppm, in Barcelona [24]), respectively. The theoretical natural carbonation rate *KnatTHEO* was used to determine the carbonation depth at 50 and 100 years (Table 10).

In Series 1, CC-I subjected to the S-process achieved the highest carbonation rate, which over a 50-year lifespan, exceeded the 20 mm minimum concrete cover defined by the standard. In addition, the RACs also achieved a carbonation depth higher than the minimum, except RAC-C50, which achieved 20 mm. However, the application of the S-C process reduced the carbonation depth of all the concretes, and the RCA concretes achieved a lower carbonation depth than the minimum concrete cover required. The RAC-C50 concrete achieved the lowest depth at 17.2 mm. The incorporation of cRCA led to lower carbonation depths compared with those observed with untreated aggregates [26]. Series 2 concretes were produced using CEM II/B and an effective w/c ratio of 0.45; the minimum concrete cover required by the standard was 25 mm. It was observed that under the S-process, the CC, RAC-C50, and RAC-C60 concretes exceeded the carbonation depth threshold of 25 mm. In contrast to the findings from Series 1, these results suggest that the incorporation of cRCA into concretes made with IIB cement did not contribute to a reduction in carbonation depth under the specified conditions, as concretes with RCA have lower depths. The presence of Ca(OH)_2_ in uncarbonated RCA can react with the pozzolans present in cements with SCM, increasing the C-S-H products and the density of cement paste [4].

As observed in Series 1, the application of CO_2_ curing (S-C process) significantly enhanced the carbonation resistance of all mixtures, resulting in reductions ranging from 12% to 21%. In all cases, the carbonation depth was reduced to below 25 mm, with RAC50-IIB exhibiting the lowest value at 18.3 mm.

## 6. Conclusions

The following conclusions can be drawn from the tests performed in this study:

- Through the accelerated carbonation process, the water absorption of RCA was reduced by 12.5%, decreasing from 6.5% to 5.6%.
- The CO_2_ curing process, conducted for 24 h at 20% CO_2_, 57% RH, and 20 °C, improved the surface of concrete specimens, guaranteeing a reduction in sorptivity capacity and a 2 mm carbonation depth.

After producing concretes using CEM I (52.5 R), with an effective w/c ratio of 0.49 (Series 1) and using CEM II/B (42.5) with an effective w/c ratio of 0.45 (Series 2), and substituting coarse NAs by 50% and 60% RCA and cRCA:

- Comparable behaviour in the fresh state was achieved in SCC incorporating NA and high percentages (50% and 60%) of RCA and cRCA.

After the concretes were subjected to the steam curing (S-process) and both steam and CO_2_ curing (S-C process) the following was achieved:

- Regarding physical properties—although CC had lower absorption than RCA and cRCA concretes, CO_2_ curing slightly improved all RACs, reducing absorption to below 4.5% with CEM I and 4.9% with CEM II.
- Regarding compressive strength—all concretes in Series 1 and 2, including CC and RAC, met the 25 MPa strength requirement after 8 h of steam curing and reached around 60 MPa at 28 days with both the S and S-C processes. RAC-C50-I and RAC-C50-IIB showed the highest strength (97% and 98% of CC strength, respectively). cRCA concretes performing slightly better than those with RCA and S-C process led to a slight strength increase at 7 and 28 days compared to the S-process.
- Regarding drying shrinkage—concretes with cRCA showed lower drying shrinkage than those with uncarbonated RCA, and CO_2_ curing further reduced shrinkage and weight loss. In Series 1, RAC-C50-I (with CEM I and cRCA) had the lowest shrinkage at 262 με, while in Series 2, despite higher shrinkage with CEM II, RAC50-II and RAC-C50 reached the lowest value of 375 με.
- Regarding durability—RACs made with cRCA showed lower sorptivity than those with uncarbonated RCA, with further reductions from CO_2_ curing—RAC-C50 and RAC-C60 in Series 1 had the lowest values (0.05 mm/min^0.5^), while RAC50 in Series 2 reached 0.06 mm/min^0.5^.
- Both RCA and cRCA improved carbonation resistance compared to CCs, and CO_2_ curing further enhanced this resistance. RAC-C50-I (Series 1) and RAC50-II (Series 2) achieved the lowest carbonation rates and an estimated 50-year lifespan, with the S-C process boosting their durability.

Overall, CO_2_ curing yielded improvements in the properties of concrete, particularly in terms of shrinkage and durability. However, when considering large-scale industrial production of this type of concrete, it is essential to account for the limitations of the technology, especially when manufacturing large precast elements. Nonetheless, the findings of this research confirm that high-performance concrete can be produced using cement with cRCA and a low clinker content, supporting efforts to lower the environmental impact of concrete production.

Given the lower performance of RAC compared to concrete made with NA, this research work clarifies that CO_2_ curing offers a sustainable solution to enhance the durability of RAC by densifying the microstructure and reducing surface permeability.

In the next phase of our research, we will analyse the microstructure of the manufactured concretes to deepen our understanding of the results achieved. We will evaluate the CO_2_ capture effectiveness of the carbonation processes and develop prototype elements to assess the real-world performance and properties of the selected concretes.

## Figures and Tables

**Table 1:** Composition of cements as a percentage of total weight.

Cement	SiO_2_	CaO	Fe_2_O_3_	Al_2_O_3_	MgO	SO_3_	Na_2_O	K_2_O	LOI
I	20.87	61.97	3.13	3.67	1.48	3.56	0.08	0.79	3.51
II/B-M	23.07	55.96	3.15	4.66	1.43	3.63	0.16	0.85	6.02

**Table 2:** Dry density and absorption capacity of aggregates.

Physical Property	FNA	CNA	RCA	CRCA
Dry Density (kg/dm^3^)	2.69	2.66	2.32	2.37
Absorption (%)	1.49	1.49	6.45	5.65

**Table 4:** Concrete specimens and test methods.

Property	Standard	Test Age (Days)	Specimen *	Specimen Size (mm)
Density and absorption	EN 12390-7 [50]	28	2	100 × 100 × 100
Compressive strength	EN 12390-3 [51]	8 h, 1, 7 and 28	2, 4, 2, 4	100 × 100 × 100
Drying Shrinkage	EN 12390-16 [52]	0–56	2	75 × 75 × 254
Sorptivity	EN ISO 15148 [53]	28	2	100 × 100 × 100
Carbonation resistance	EN 12390-12 [54]	7, 28, 56	2	100 × 100 × 200

* Number of specimens used in each of the S and S-C processes.

**Table 5:** Sorptivity and carbonation depth used to determine CO_2_ curing time.

Mix/Curing Process	Sorptivity (mm/min^0.5^)		Carbonation Depth (mm)	
	(S-1dC)	(S-2dC)	(S-C 1d)	(S-C 2d)
CC-I	0.0490	0.0493	2.0	3.6
RAC-C50-I	0.0538	0.0456	2.0	3.8
CC-IIB	0.0571	0.0405	2.7	3.2
CRCA50-IIB	0.0659	0.0565	3.0	4.2

**Table 6:** Physical properties and sorptivity on Day 28 days of concrete production.

Mix/Curing Process	Dry Density (Kg/dm^3^)		Absorption (%)		Accesible Porosity (%)		Sorptivity (mm/min^0.5^)	
	(S)	(S-C)	(S)	(S-C)	(S)	(S-C)	(S)	(S-C)
Series 1								
CC-I	2.41	2.41	3.54	3.61	8.55	8.70	0.087	0.055
RAC50-I	2.34	2.35	3.99	3.87	9.33	9.10	0.094	0.066
RAC60-I	2.32	2.33	4.26	4.18	9.90	9.75	0.093	0.060
RAC-C50-I	2.33	2.34	4.67	4.44	10.87	10.41	0.088	0.053
RAC-C60-I	2.34	2.34	4.26	4.12	9.94	9.65	0.075	0.045
Series 2								
CC-IIB	2.36	2.37	4.41	4.06	10.42	9.63	0.099	0.066
RAC50-IIB	2.30	2.30	5.21	4.95	12.01	11.35	0.112	0.062
RAC60-IIB	2.31	2.32	4.38	4.23	10.13	9.83	0.114	0.081
RAC-C50-IIB	2.33	2.33	4.51	4.44	10.50	10.34	0.107	0.081
RAC-C60-IIB	2.30	2.31	5.13	4.61	11.78	10.62	0.120	0.082

**Table 7:** Compressive strengths of all concretes at 8 h, 1 day, and 28 days (in brackets, the standard deviation).

Concrete Type	Compressive Strength (MPa)					
	8 h	1 d	7 d		28 d	
	S	S	S	S-C	S	S-C
CC-I	29.8 (0.2)	37.4 (3.9)	56.7 (1)	57.5 (3.2)	61.3 (2.5)	65.9 (3.8)
RAC50-I	27.6 (0.7)	34.2 (1.1)	51.8 (1.7)	52.7 (2.2)	58.6 (2.2)	59.9 (1.4)
RAC60-I	28.4 (2.3)	34.1 (1.4)	49.9 (0.5)	53.2 (0.1)	57.8 (1.5)	60.0 (1.5)
RAC-C50-I	30.9 (0.2)	37.4 (1.2)	52.2 (0.5)	55.4 (1.5)	62.9 (0.6)	64.1 (1.0)
RAC-C60-I	28.0 (0.5)	35.1 (0.5)	50.3 (2.1)	52.1 (0.5)	59.7 (1.5)	60.4 (1.8)
CC-IIB	28.1 (2.2)	35.5 (1.6)	52.0 (0.3)	53.5 (0.5)	61.7 (1.9)	62.5 (3.9)
RAC50-IIB	27.8 (0.3)	34.7 (0.3)	48.3 (0.1)	50.1 (0.3)	58.8 (1.9)	59.7 (2.4)
RAC60-IIB	30.5 (1.7)	33.8 (2.8)	51.6 (0.7)	52.4 (0.2)	59.1 (1.0)	59.8 (0.6)
RAC-C50-IIB	28.5 (1.2)	34.3 (1.8)	51.2 (0.8)	54.2 (0.6)	60.2 (0.8)	61.6 (0.6)
RAC-C60-IIB	26.2 (0.1)	32.8 (0.7)	50.2 (0.4)	50.9 (0.8)	58.7 (0.9)	61.2 (1.3)

**Table 8:** Shrinkage values and mass loss for all the concrete specimens after 56 days.

Mix/Curing Process	Shrinkage (με) 56d			Mass Loss (%)	
	(S)	(S-C)	Variation (%)	(S)	(S-C)
Series 1					
CC-I	−335	−264	21	2.4%	2.1%
RAC50-I	−451	−413	8	3.1%	2.8%
RAC60-I	−335	−317	5	3.3%	2.7%
RAC-C50-I	−262	−274	−5	2.8%	2.5%
RAC-C60-I	−344	−321	7	3.3%	2.9%
Series 2					
CC-IIB	−364	−348	4	2.5%	2.0%
RAC50-IIB	−413	−372	10	3.0%	2.3%
RAC60-IIB	−478	−443	7	3.0%	2.9%
RAC-C50-IIB	−352	−378	−7	3.1%	2.9%
RAC-C60-IIB	−449	−443	1	3.3%	2.9%

**Table 9:** Carbonation depth after 70 days for Series 1 and Series 2 concretes.

Concrete Reference	Series 1 Cem I				Series 2 Cem IIB			
	Carbonation Depth (mm) at 70 Days		*kacc* (mm/dia^0.5^)		Carbonation Depth (mm) at 70 Days		*kacc* (mm/dia^0.5^)	
	(S)	(S-C)	(S)	(S-C)	(S)	(S-C)	(S)	(S-C)
CC	14.61	13.48	1.64	1.34	13.79	13.02	1.57	1.31
RAC50	13.41	12.33	1.54	1.20	13.08	11.57	1.44	1.14
RAC60	12.84	12.81	1.48	1.25	12.95	12.52	1.46	1.27
RAC-C50	11.73	10.97	1.28	1.07	13.84	13.03	1.56	1.37
RAC-C60	12.80	11.20	1.42	1.12	14.25	13.23	1.58	1.28

**Table 10:** Theoretical natural carbonation rate and estimated carbonation depth over a 50- and 100-year lifespan.

Concrete Reference	Serie 1 Cem I						Serie 2 Cem IIB					
	Carbonation Coefficient.		Carbonation Depth (mm)				Carb. Coeffic.		Carbonation Depth (mm)			
	*KnatTHEO* (mm/Year^0.5^)		50 Years		100 Years		*KnatTHEO* (mm/Year^0.5^)		50 Years		100 Years	
	(S)	(S-C)	(S)	(S-C)	(S)	(S-C)	(S)	(S-C)	(S)	(S-C)	(S)	(S-C)
CC	3.8	3.1	26.4	21.7	37.3	30.7	3.6	3.0	25.3	21.1	35.8	29.8
RAC50	3.5	2.7	24.9	19.4	35.2	27.4	3.3	2.6	23.3	18.4	32.9	26.0
RAC60	3.4	2.9	23.9	20.2	33.8	28.5	3.4	2.9	23.7	20.6	33.5	29.0
RAC-C50	2.9	2.5	20.1	17.3	29.3	24.5	3.5	3.1	25.2	22.2	35.6	31.4
RAC-C60	3.2	2.6	23.0	18.4	32.5	26.1	3.6	2.9	25.6	20.6	36.1	29.2

## Data Availability

The original contributions presented in this study are included in the article. Further inquiries can be directed to the corresponding author.
